# Non-specific yet selective interactions contribute to small molecule condensate partitioning behavior

**DOI:** 10.21203/rs.3.rs-4784242/v1

**Published:** 2024-08-13

**Authors:** Bin Zhang, Cong Wang, Henry Kilgore, Andrew Latham

## Abstract

Biomolecular condensates are essential in various cellular processes, and their misregulation has been demonstrated to be underly disease. Small molecules that modulate condensate stability and material properties offer promising therapeutic approaches, but mechanistic insights into their interactions with condensates remain largely lacking. We employ a multiscale approach to enable long-time, equilibrated all-atom simulations of various condensate-ligand systems. Systematic characterization of the ligand binding poses reveals that condensates can form diverse and heterogeneous chemical environments with one or multiple chains to bind small molecules. Unlike traditional protein-ligand interactions, these chemical environments are dominated by non-specific hydrophobic interactions. Nevertheless, the chemical environments feature unique amino acid compositions and physicochemical properties that favor certain small molecules over others, resulting in varied ligand partitioning coefficients within condensates. Notably, different condensates share similar sets of chemical environments but at different populations. This population shift drives ligand selectivity towards specific condensates. Our approach can enhance the interpretation of experimental screening data and may assist in the rational design of small molecules targeting specific condensates.

## Introduction

Biomolecular condensates are membrane-less organelles that play critical roles in organizing the intracellular environment, facilitating the spatial and temporal regulation of biochemical processes.[1–4] Their formation is driven by multivalent interactions among specific proteins and nucleic acids, leading to the establishment of dynamic and reversible assemblies.[5–11] These condensates can rapidly form and dissolve in response to cellular signals or changes in the cellular environment, allowing cells to adapt to various physiological conditions efficiently.[12–14]

Dysregulation of condensates can lead to various diseases, sparking interest in discovering molecules that target them for new therapeutic approaches.[15–19] Changes in the formation or dissolution of these condensates can contribute to pathological conditions such as neurodegenerative diseases, cancers, and genetic disorders.[16,18,20–25] Developing chemical probes and therapeutics that can modify the properties of condensates offers a promising strategy for studying and potentially treating these conditions. Small molecules may help restore normal cellular functions in diseases where condensate localization, dynamics, and material properties are altered.[14,16,19,26,27]

Initial progress has been made toward understanding the interactions between condensates and ligands, with several groups conducting large-scale screening studies.[19,28–30] These studies quantify the fraction of small molecules inside the condensate versus those outside, referred to as partition coefficients, using various techniques. Several trends emerge across the library of molecules investigated. Small molecule mass spectrometry experiments have led to the discovery that lipids and small molecule drugs can concentrate in condensates, as a result of their hydrophobic character.[29,30] Fluorescent probe partitioning experiments explored using microscopy has[14,28] uncovered differences in the chemical environments of condensates that could be predictive of small molecule partitioning behavior in live cells. Insights into the atomistic interactions driving small molecule partitioning could help understand the reported results and guide the rational design of molecules that could interact with condensate.

Protein-ligand interactions have been extensively studied, primarily focusing on single folded proteins.[31–34] However, the unique properties of condensates may result in interactions with ligands that differ from established paradigms. Notably, many proteins that form condensates are disordered, lacking the well-folded regions necessary for creating binding pockets to facilitate specific interactions.[11,15,35,36] Additionally, condensates consist of multiple chains, raising the question of whether cross-chain interactions contribute to ligand binding differently than in single chains. Molecular dynamics (MD) simulations, which have been instrumental in identifying key factors for ligand binding affinity and in the rational design of small molecules targeting single proteins,[33,37,38] can also be applied to study condensate-ligand interactions.

We conduct long-timescale atomistic MD simulations to reveal the chemical environments within protein condensates that facilitate the partitioning of ligand molecules. A multiscale approach was used to equilibrate protein-protein contacts in three models of cellular condensates modeled by homotypic protein condensates: transcriptional (MED1), granular cluster of the nucleolus (NPM1), and constitutive heterochromatin (HP1$\alpha$), which have been previously characterized experimentally.[19] We introduce protein-ligand interaction fingerprints to systematically characterize the variety of binding poses observed in the atomistic simulations. Low-dimensional embedding of these interaction fingerprints revealed multiple clusters of chemical environments for ligand binding. These environments feature nonspecific interactions, often enriched with hydrophobic residues from both the same and different protein chains, highlighting the significant role of ligand hydrophobicity in determining partition coefficients. Notably, the simulations identified specific chemical groups in ligand molecules that contribute to these interactions. Ligands enriched with these groups were found to partition more effectively in protein condensates compared to those without them. Microenvironments with similar chemical compositions were found across the three protein condensates, indicating common features. However, significant shifts in the population of these chemical environments were observed, contributing to the selectivity of ligands targeting specific condensates.

## Results

### Multiscale simulations produce consistent protein interactions across resolutions

Computer simulations have been instrumental in characterizing the detailed structural organization of condensates and linking their stability and material properties to sequence features.[39–50] These simulations often employ coarse-grained models due to their superior computational efficiency, which is essential for ensuring the equilibration of complex, many-chain systems.[40,42,44,51] However, coarse-grained models lack the atomistic details necessary to capture the interactions and solvation environment critical for ligand binding.

To obtain equilibrium configurations of condensates with well-relaxed inter-chain contacts while preserving atomistic details of protein-ligand interactions, we employed a multiscale approach.[52–54] As described in the *Methods* section and illustrated in Figure 1A, this approach involves three steps that gradually increase the model resolution. We began with an $\alpha$-carbon-only protein model, MOFF,[40,55] that facilitates large-scale conformational changes. Next, we transitioned to an explicit solvent coarse-grained model with the MARTINI force fields to relax the solvation environment around proteins.[56] Finally, we employed an explicit solvent all-atom model with the CHARMM36m force field[57] for accurate descriptions of protein-ligand interactions.

We applied the multiscale approach to construct all-atom models for three condensates that have been extensively characterized in prior experiments: HP1$\alpha$, MED1, and NPM1. Simulations at different resolutions produced consistent interchain contact patterns among various amino acids (Figures 1B and S1-S3). $R^2$ values between the MOFF or MARTINI and all-atom simulation results are above 0.75 for all proteins. We also repeated the simulation

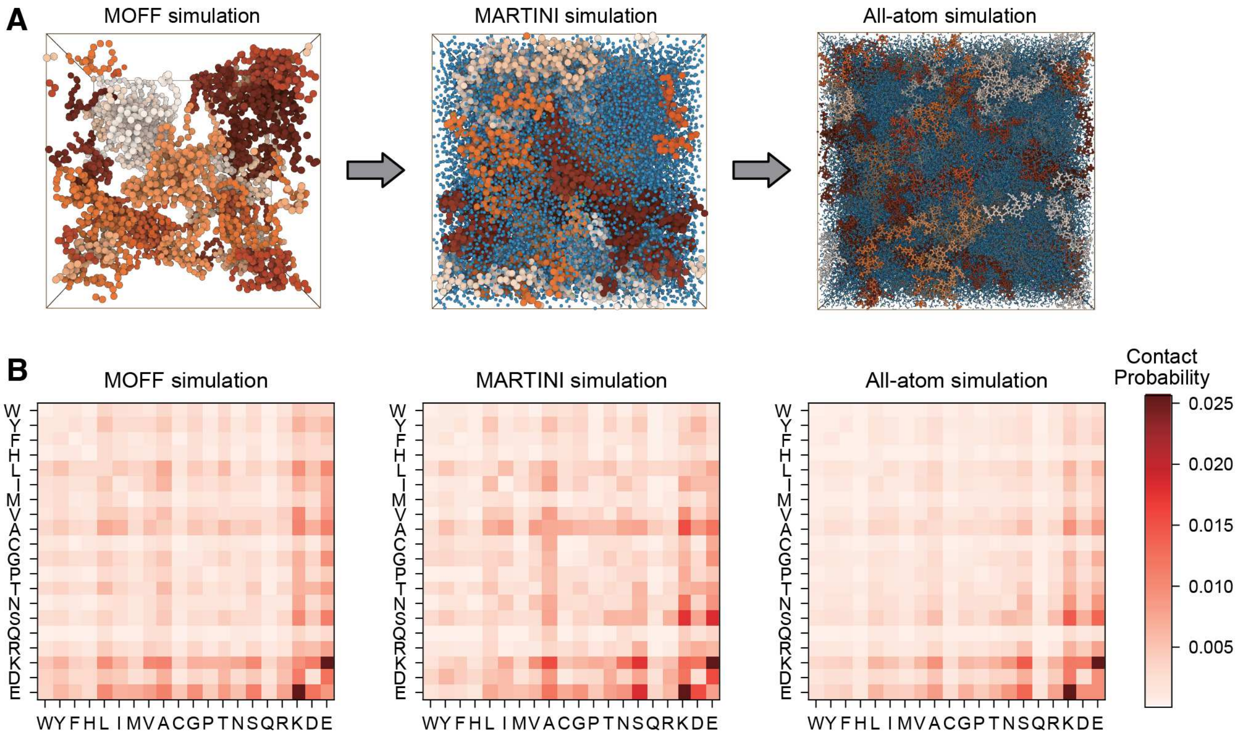

Figure 1: **Multiscale approach enables explicit solvent all-atom simulations of biomolecular condensates with equilibrated configurations.** (A) Illustration of the multiscale simulation protocol that gradually increases the model resolution. (B) Contact probability maps for amino acids from different protein chains, computed from simulations at various resolutions, exhibit consistent patterns.

protocol for HP1$\alpha$ three times, each time selecting different configurations produced by MOFF simulations to initialize the higher-resolution simulations. The resulting three all-atom simulations yielded similar statistics for amino acid contacts (Figure S4). These results support the multiscale approach's ability in producing equilibrated, atomistic condensate organizations.

### Interaction fingerprints reveal the chemical and dynamical nature of condensate-ligand interactions

Using the atomistic configurations derived from multiscale simulations, we conducted additional two-microsecond explicit solvent atomistic MD simulations to investigate the interactions between protein condensates and small molecules. We focused on three types of small molecules: Mitoxantrone, Daunorubicin, and Proflavine, which have been shown to differentially partition in the three condensate systems.[28] For HP1$\alpha$ condensates, we also simulated a more hydrophilic molecule, Citicoline. A total of 10 simulations were performed, each corresponding to a specific condensate-ligand combination, resulting in a cumulative simulation time of 20 microseconds.

The condensate systems consist of numerous protein copies, creating a wide variety of local chemical environments. To comprehensively characterize all potential chemical environments that could accommodate small molecules, we introduced 80 copies of each small molecule into the condensate system for simulations. The presence of multiple small molecules enabled the exploration of potential binding poses in parallel, rather than relying on the slower diffusion process of individual small molecules.

To systematically characterize the numerous binding environments identified in simulations, we adopted the chemical interaction fingerprint concept, commonly used for encoding protein-ligand interactions.[58–62] As detailed in the *Methods* section and illustrated in Figure 2A, these fingerprints represent each binding pose with a binary vector that annotates the sets of amino acids contacting the ligand molecule and the types of interactions formed. Consequently, the fingerprints capture both structural and chemical information of the binding pose. To simplify fingerprint representation, we consolidated amino acids from different chains. When preserving the residue index of each amino acid during this consolidation, we refer to the resulting vector as index-based fingerprints. However, unless otherwise specified, we further group amino acids of the same type together without preserving the residue indices to facilitate transferable analyses across proteins.

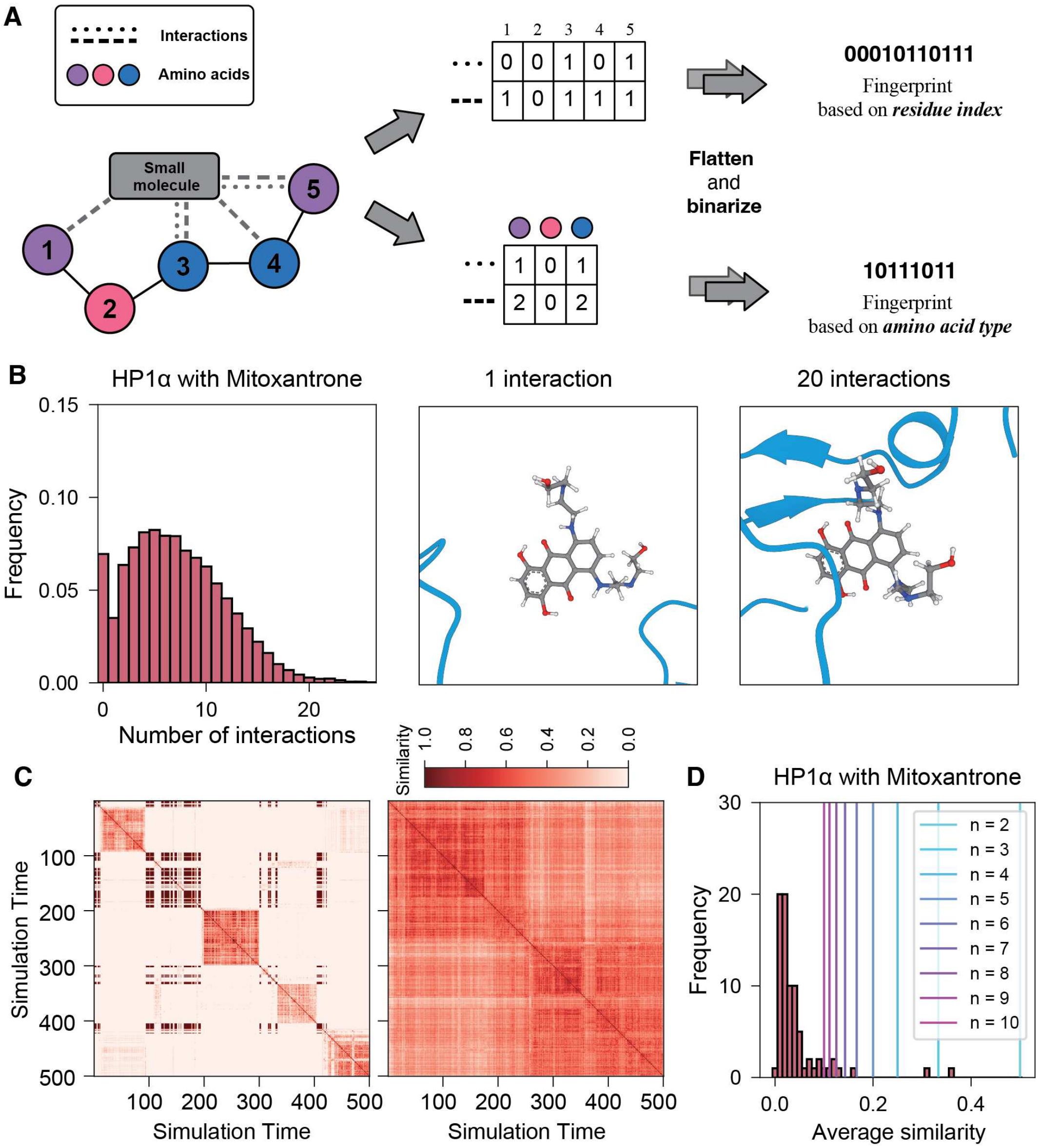

Figure 2: **Chemical interaction fingerprints facilitate the analysis of condensate-ligand interactions.** (A) The diagram illustrates the definition of the interaction fingerprint, which characterizes interactions between a small molecule and the set of contacting amino acids. Two types of fingerprints can be distinguished based on whether residue indices or types are preserved when consolidating amino acids from different chains. (B) Probability distribution of the total number of interactions detected in all binding poses uncovered from simulations of the HP1$\alpha$ condensate with Mitoxantrone. Representative configurations depicting condensate-ligand binding with a small and large number of interactions are shown on the side. (C) Representative similarity matrices of fingerprints along the simulation trajectory, corresponding to a dynamic ligand traversing through multiple binding sites (left) and a stably bound ligand (right). (D) Probability distribution of the average of similarity matrices for different Mitoxantrone molecules. The vertical lines denote theoretical lower limits of this average for a ligand exploring $n$ unique binding poses.

The statistics of interaction fingerprints revealed the diversity of small molecule binding poses. For example, we calculated the probability distribution of the total number of interactions, determined as a direct sum of the fingerprint vector, formed between each Mitoxantrone molecule and the HP1$\alpha$ condensate. As shown in Figure 2B, the number of interactions in the binding poses varies widely. The average number of amino acids involved in ligand binding is approximately five (Figure S6). This diversity in binding poses supports the non-specific nature of condensate-ligand interactions. Analyses of other systems yield similar results (Figures S5 and S6). Furthermore, the statistical similarity between the first and second halves of the trajectories supports the convergence of the simulations (Figures S7 and S8).

To elucidate the dynamic nature of condensate-ligand interactions, we computed the similarity of index-based fingerprints for each ligand molecule at different simulation times (Eq. 1 in the *Methods* section). The similarity values range from 0, indicating no overlap between two binding poses, to 1, indicating identical binding poses (see *Methods* for details). The similarity values along a simulation trajectory can be represented as a matrix, providing a visual representation of ligand dynamics (Figures 2 and S9). When the ligand interacts with multiple distinct binding sites, high similarity values appear during the time it occupies each site, forming a domain along the diagonal in the corresponding matrix (Figure 2C, left). Off-diagonal elements, representing similarity values for fingerprints in different binding sites, are low. In contrast, if a ligand remains stably bound to a single site, the similarity matrix displays high values throughout (Figure 2C, right).

The average of the similarity matrix indicates the number of binding poses explored by the ligand, particularly when compared with theoretical values estimated for ligands binding at exactly $n$ sites during the simulation (see *Supporting Information* for details). As shown in Figure 2D, for most ligands, the similarity matrix average is lower than the theoretical value calculated for $n = 10$. This suggests that ligand molecules dynamically explore numerous distinct binding environments within the condensate. Similar conclusions can be drawn from analyses of other condensate-ligand systems (Figure S10).

### Chemical environments are created from single and multichain interactions

While protein-ligand interactions have been extensively studied,[31–34] condensates, which are assemblies of interacting proteins and nucleic acids, exhibit emergent properties distinct from a single biopolymer. Understanding the role of cross-chain contacts in small molecule partitioning could be crucial for differentiating condensate-drug interactions from those occurring in single proteins. We again focus our analysis on the HP1$\alpha$ condensate, with results for other systems provided in the *Supporting Information* (Figures S11-S13).

To determine whether small molecules interact with a single or multiple chains during binding, we introduced the concept of single-chain dominance ($f_{\mathrm{SCD}}$, Eq. 2 in the *Methods* section). This metric quantifies the maximum fraction of interactions in a binding pose originating from a single chain. By definition, $f_{\mathrm{SCD}}$ ranges from 0 to 1, reaching 1 if the small molecule interacts exclusively with one protein chain. We further determined the probability distribution of $f_{\mathrm{SCD}}$ using binding poses from simulations. As shown in Figure 3A, a significant fraction of the binding poses have a non-unity $f_{\mathrm{SCD}}$, indicating the involvement of multiple protein chains in small molecule interactions.

The presence of both single and multiple chain environments prompted us to examine the types of interactions they offer for ligand binding. We performed Uniform Manifold Approximation and Projection (UMAP) embedding analysis to project the high-dimensional interaction fingerprints onto two variables, UMAP1 and UMAP2. As detailed in the *Methods* section, UMAP is a dimension reduction technique that finds a low-dimensional projection of the data that best preserves their topological structure.[63] The UMAP embeddings succeeded in partitioning the interaction fingerprints into several clusters (Figure 3D). We observed a significant overlap between the fingerprints from a single protein and those involving multiple chains. This observation is robust across different small molecules and condensates (Figure

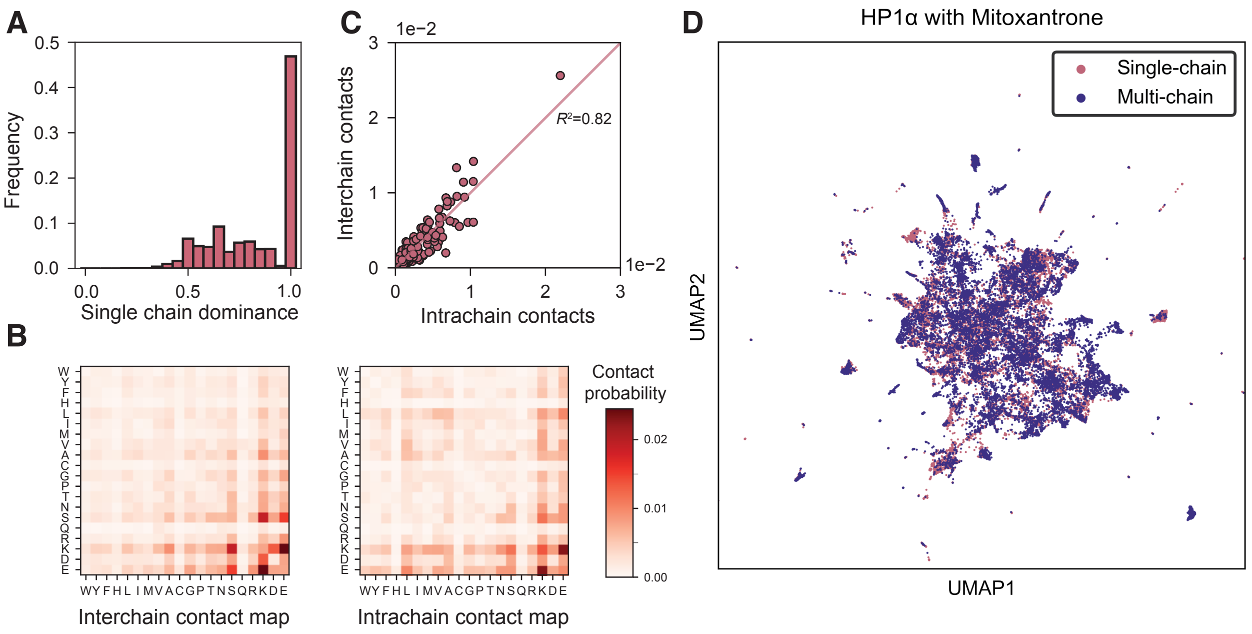

Figure 3: **Amino acids from single or multiple protein chains form scaffolds with similar chemical environments for ligand binding.** Results are obtained from simulations of the HP1$\alpha$-Mitoxantrone system. (A) Probability distribution of the single chain dominance. (B) Contact maps between amino acids from different (left) and the same (right) chain. (C) Correlation between the intra- and inter-chain amino acid contacts. The red line represents a linear fit to the data. (D) Scatter plot of the interaction fingerprints on the two dimensional UMAP embeddings. Binding poses with single and multiple chains are shown in pink and blue, respectively.

S12). Thus, while amino acids from different protein chains can come together to bind small molecules, the chemical environments they offer are similar to those found in individual proteins.

The similarity in binding environments can be inferred from the contact maps between amino acids from the same and different chains (Figure 3B). The two maps show a significant correlation, with a $R^2$ value of 0.82 (Figure 3C). Consequently, the scaffold for condensate-ligand binding shares a similar amino acid composition, regardless of whether it is formed by a single protein chain or multiple chains, resulting in comparable chemical environments and interaction fingerprints.

### Multiple chemical environments contribute to ligand partitioning

The interaction fingerprints enable a systematic analysis of the chemical nature of the binding between small molecules and condensates. To this end, we computed the fraction of different types of interactions detected. As shown in Figures 4A and S14, non-specific interactions, such as hydrophobic interactions and van der Waals contacts, are important for interactions between the ligands and proteins considered. Furthermore, as the ligands become more hydrophobic, as measured by their $\log P$ (the logarithm of the partition coefficient of a molecule between octanol and water), the fraction of hydrophobic interactions increases.

To further distinguish among various small molecules, we utilized UMAP to project their interaction fingerprints detected in the HP1$\alpha$ condensate onto two dimensions (Figure 4B and S15). Similar results for other condensate-ligand systems are provided in Figures S16 and S17. The embeddings segregate the fingerprints into minimally overlapping clusters, each containing unique combinations of amino acids for ligand binding. Figure 4C demonstrates that while clusters 1 and 4 are enriched with either hydrophilic or hydrophobic residues, clusters 2 and 3 comprise a mixture of both residue types.

We found that all four molecules are present in most clusters, albeit in varying proportions. The lack of clear separation among the small molecules aligns with the dominance of non-specific interactions provided by condensates for binding. The shift in the population of binding environments is most evident in the separate scatter plots for each drug, with the fingerprints colored by the fraction of hydrophobic interactions (Figure 4D). Most fingerprints for the hydrophilic molecule Citicoline are located around cluster 1. In contrast, fingerprints for the hydrophobic molecule Proflavine are concentrated around cluster 4.

Thus, condensates offer heterogeneous chemical environments for non-specific ligand binding. Depending on their hydrophobicity, the small molecules preferentially engage with subsets of chemical environments that provide favorable interactions. The importance of drug hydrophobicity in condensate binding is consistent with previous findings on its role in predicting partitioning coefficients.[29,64]

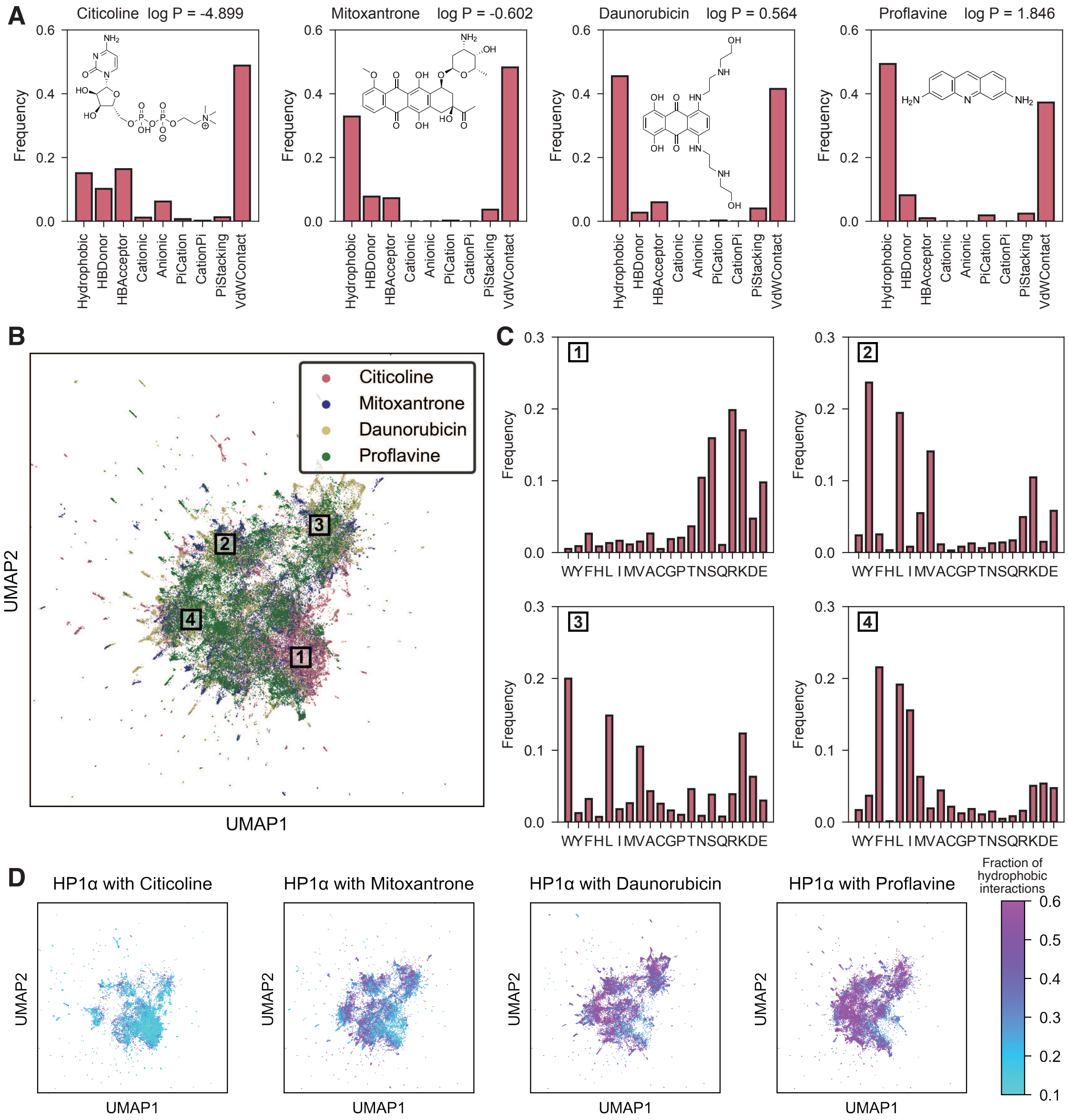

Figure 4: **Condensates provide heterogeneous chemical environments for non-specific ligand binding.** Results from the HP1$\alpha$ condensate are shown as examples. (A) Frequency of various interactions detected in the binding pose between HP1$\alpha$ and different small molecules. (B) Scatter plot of the interaction fingerprints for the four small molecules in the HP1$\alpha$ condensate on the two dimensional UMAP embeddings. (C) Amino acid frequency determined from the interaction fingerprints that fall into the four regions with distinct UMAP embeddings shown in part B. (D) Scatter plot of the same UMAP embedding as in part B but shown separately for different small molecules. Interaction fingerprints are color-coded according to their fraction of hydrophobic interactions.

### Simulations identify small molecule fragments contributing to condensate partitioning

In addition to the condensate chemical environments, the atomistic simulations also revealed the chemical groups of small molecules that interact with amino acids forming such environments (Figure 5A). While the chemical properties of these fragments varied among the small molecules, they shared some common moieties (e.g., amines, $\pi$-systems, and carbonyl groups) that are expected to drive noncovalent interactions with proteins (Figure 5A). These fragments provide direct predictions that can be tested against experimental screening data: small molecules enriched in these moieties are expected to partition more effectively in condensates than those lacking them.

Analysis of the screening data in Kilgore et al.[28] revealed that several interaction fragments derived from Mitoxantrone, Daunorubicin, and Proflavine were identifiable in certain chemical probes (Figure 5B). Comparing the partition ratios of probes containing one of these interaction fragments (+) for specific condensates (MED1, NPM1, HP1$\alpha$) with those lacking the fragment showed that partitioning behavior was influenced by its presence (Figure 5B). For instance, chiral secondary alcohols were equally incorporated and weakly associated with partitioning. Secondary amines adjacent to a $\pi$-system had minimal impact on the partition ratio in MED1 condensates but were more influential in NPM1 and HP1$\alpha$ condensates. Aromatic secondary amines were less likely to concentrate in MED1 condensates but had a significant impact in NPM1 and HP1$\alpha$ condensates. These results further support the idea that chemical environments in condensates enable selective small molecule partitioning and MD simulation can help guide the discovery of molecules that favor them.

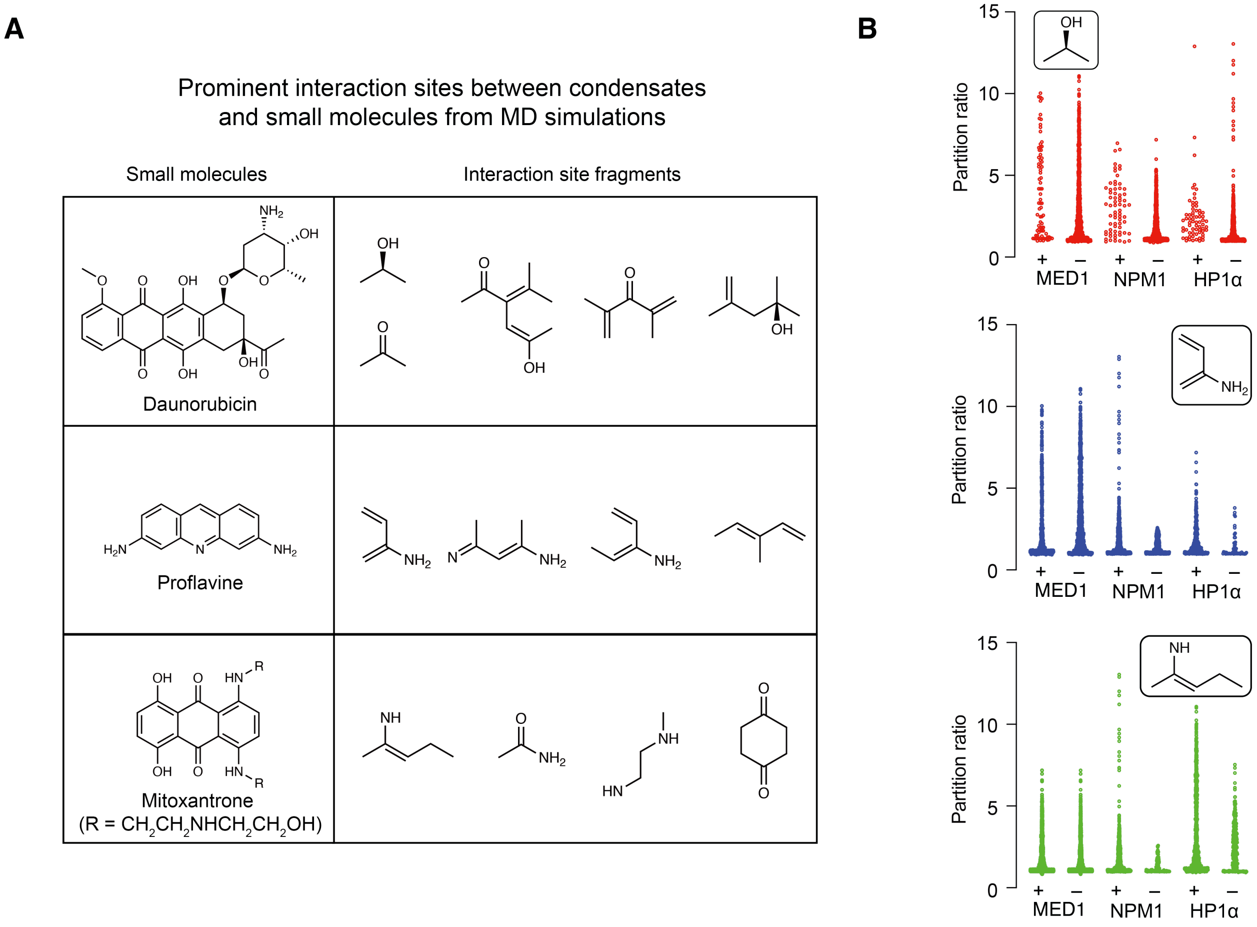

Figure 5: **MD simulations identify small molecule interaction fragments that facilitate condensate partitioning.** (A) Interacting fragments of small molecules identified from atomistic simulations. (B) Experimental partitioning ratios of drugs with/without fragments in panel A.

### Population shift in chemical environments contributes to differential ligand partitioning

Having revealed the heterogeneous chemical environments individual condensates harbor to accommodate small molecules, we next examined the distinctions of these interactions across different condensates for the same molecule.

We applied UMAP to analyze interaction fingerprints formed between Mitoxantrone and the three different protein condensates. Similar to those presented in Figure 4, the embeddings successfully identified the heterogeneous environments within condensates, with

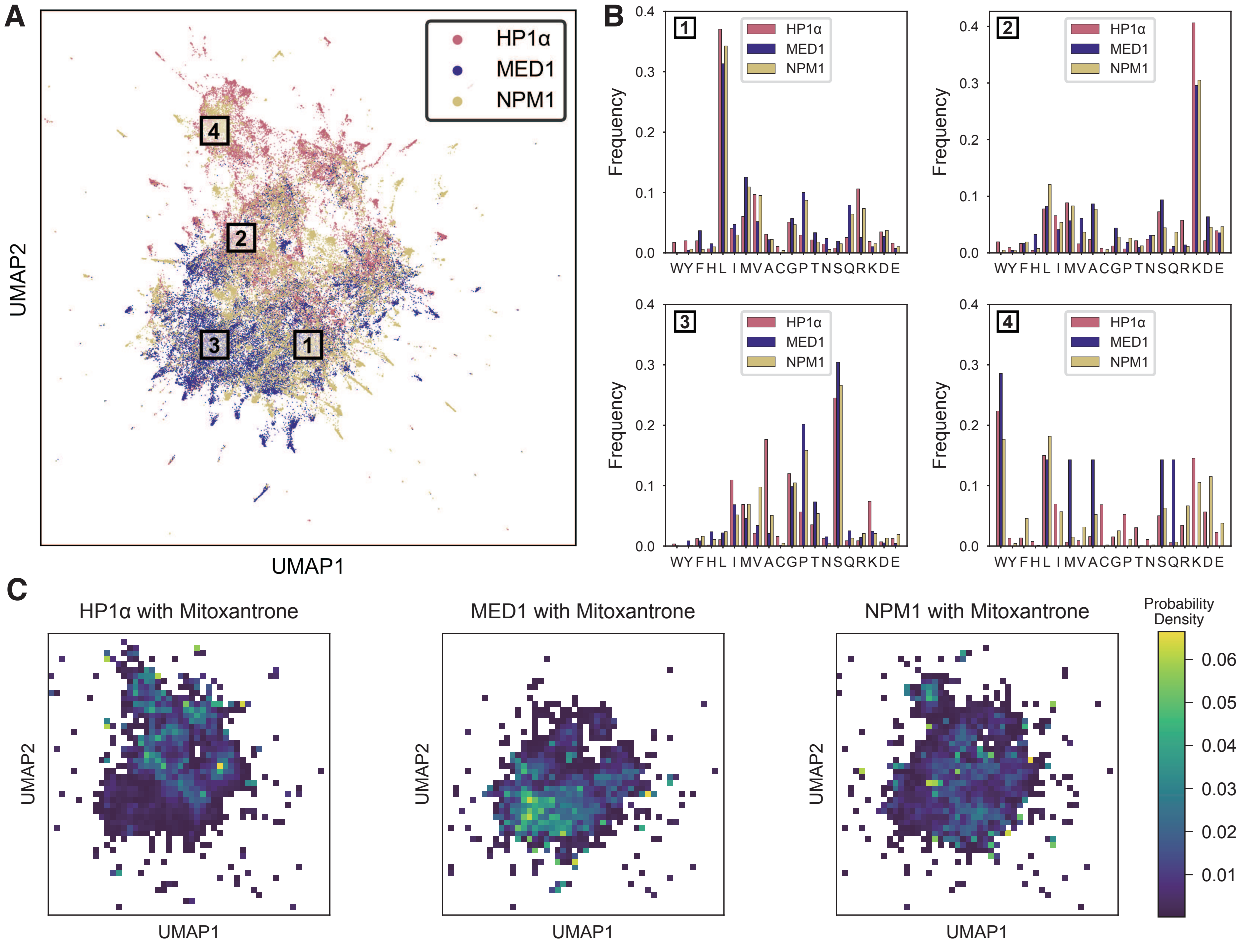

Figure 6: **Mitoxantrone identifies similar chemical environments for contact across three protein condensates.** (A) Scatter plot of the interaction fingerprints for Mitoxantrone in the three condensate. (B) Amino acid frequency determined from the interaction fingerprints that fall into the four regions with distinct UMAP embeddings shown in part A. (C) Probability density profile of the interaction fingerprints for Mitoxantrone in the three condensate.

visible clusters of interaction fingerprints shown in Figure 6A. Notably, the scatter plots of the binding modes from the three condensates exhibit significant overlap. Analyses of the other two molecules produced similar results (Figures S18 and S19). Thus, small molecules can concentrate in similar chemical environments found in different condensates. The amino acid composition in different clusters is highly conserved across the three condensates (Figure 6B).

Although the interaction fingerprints from various condensates exhibit considerable overlap, their relative distribution within the embedding space differs markedly. To examine these quantitative differences, we plotted the probability densities of interaction fingerprints in Figure 6C. The peaks of the distributions (yellow) are located at distinct positions. Furthermore, we found that the frequency of amino acids in the binding poses correlates with the frequency computed directly from the protein sequence (Figure S20). Therefore, while condensates share similar chemical environments to accommodate small molecules, they fine-tune the relative population with amino acid composition.

The above observations enhance our understanding of experimental data regarding the correlation of small molecule partitioning coefficients in different protein condensates. Given the similar chemical environments of various protein condensates, small molecules that are favored in one are likely to partition in others. However, variations in the relative distribution of chemical environments lead to quantitative differences in partition coefficients across condensates.

## Conclusions and Discussion

In this study, we employed a multiscale simulation approach to investigate condensate-ligand interactions at atomic resolution. We also introduced a quantitative metric, the interaction fingerprint, to systematically characterize the chemical and structural features of the chemical environment for ligand binding. Analysis of these fingerprints revealed that different condensates utilize remarkably similar chemical environments for ligand binding, explaining the correlation of small molecule partitioning coefficients across condensates. However, these microenvironments are quite heterogeneous, comprising diverse binding pockets formed by distinct amino acid groups. Variations in the distribution of these pockets contribute to condensates' preference for specific ligand molecules, resulting in the observed ligand selectivity.

The similarity in chemical environments across condensates arises from the shared driving forces that contribute to condensate organization. Previous studies have shown that most condensates rely on hydrophobic, electrostatic, and cation-$\pi$ interactions to mediate contacts between disordered protein chains.[5–10,65] These interactions promote interchain contacts with similar amino acid compositions across condensates. Since small molecules only passively interact with the preformed protein-interaction network by different chains, they encounter similar chemical environments for binding.

## Methods

### Multiscale simulation of condensates

We employed a multiscale approach to prepare equilibrated configurations of protein condensates to investigate ligand binding environments. Initially, we used coarse-grained simulations with MOFF, a one-bead-per-amino-acid force field, which balances interactions for both folded and disordered proteins.[40] Simulations were initialized by randomly placing protein molecules, 10 for HP1$\alpha$ dimers, 14 for NPM1, and 4 for MED1, in a cubic box of 100 nm $\times$ 100 nm $\times$ 100 nm. The process began with energy minimization followed by 0.1 $\mu$s constant pressure and constant temperature (NPT) simulations at 150 K and 1 bar, using a 10 fs timestep. This resulted in the compression of the simulation boxes to a final size of of 15 $\times$ 15 $\times$ 15 nm$^3$. The box size was chosen to achieve a protein mass density of approximately 130 mg/mL, consistent with previous studies of protein condensates.[40,43,44,66] Subsequent energy minimizations were followed by 0.1 $\mu$s constant volume and constant temperature (NVT) simulations, with a 10 fs timestep. During the NVT simulation, the temperature was gradually increased from 150 K to 300 K using the GROMACS[67] simulated annealing protocol. Finally, NVT simulations were conducted at 300 K for 2 $\mu$s with a 10 fs timestep.

Following these simulations, we used the MARTINI force field,[68] which includes explicit representations for water molecules and ions, and higher resolution representations for proteins based on a four heavy atoms to one coarse-grain bead mapping strategy. The final frames from the MOFF simulations were converted to atomic structures using REMO,[69]

and then coarse-grained using Martinize2.[70] The MARTINI protein condensates were solvated with coarse-grained water molecules and NaCl ions at a concentration of 150 mM, achieving a box size of 15 × 15 × 15 $nm^3$. MARTINI simulations began with energy minimization, followed by NVT equilibration at 313.15 K for 5 ns with a 10 fs timestep, and NPT equilibration at 313.15 K and 1 bar for 10 ns with a 20 fs timestep. NPT production simulations were then conducted at 313.15 K and 1 bar for 0.6 $\mu$s with a 20 fs timestep.

Condensate structures from the final frames of the MARTINI simulations were converted to atomic structures using the Backward tool[71] for simulations with the CHARMM36m force field. The protein condensates were solvated with CHARMM-modified TIP3P water and 150 mM NaCl to achieve a box size of 15 × 15 × 15 $nm^3$. Before introducing drug molecules, we equilibrated the protein-only system with a 125 ps NVT simulation at 313.15 K using a 2 fs timestep, followed by a 125 ps NPT simulation at 313.15 K and 1 bar with a 2 fs timestep. We then conducted a 500 ns NPT simulation at 313.15 K and 1 bar with a 4 fs timestep.

After equilibration, 80 small-molecule drugs were inserted into the system by randomly replacing solvent molecules using the GROMACS `insert-molecules` tool, achieving a concentration of approximately 40 mM. Force fields for these molecules were generated using CGenFF.[72,73] We performed 2 $\mu$s NPT simulations at 313.15 K and 1 bar with a 4 fs timestep to probe protein-ligand interactions. For all atomistic simulations with a 4 fs timestep, bond constraints were applied using the LINCS algorithm,[74] and the hydrogen mass repartitioning method[75] was used.

All simulations were performed using GROMACS,[67] with additional simulation details provided in the *Supporting Information*. System visualization and analysis were conducted with Mol*,[76] MDAnalysis,[77] and MDTraj.[78]

### Construction of condensate-ligand interaction fingerprints

We utilize the Structural Interaction Fingerprint (SIFt) methodology as outlined in previous studies[58] to characterize condensate-ligand interactions via the Python library Pro-

LIF.[62] Initially, we construct an interaction matrix $\mathbf{M}$ with dimensions $N \times L$ for a given condensate-ligand conformation. Here, $N = 6$ represents the number of interaction types considered, which include `Hydrophobic`, `HBDonor`, `HBAcceptor`, `PiCation`, `PiStacking`, and `VdWContact`. The parameter $L = n \times l$ where $l$ denotes the number of amino acids in a protein chain, and $n$ indicates the number of proteins in the condensate. Each element $M_{i,j}$ in the matrix is binary (1 or 0), indicating the presence or absence of the $i$-th interaction type between the ligand and the $j$-th residue.

We reduce the dimensionality of the interaction matrix by summing the columns corresponding to different protein chains but sharing the same sequence location, producing a matrix $\mathbf{M}_{\text{index}}$ with dimensions $6 \times l$. Additionally, we define another matrix, $\mathbf{M}_{\text{AA}}$, with dimensions $6 \times 20$ by merging all columns of the same amino acid type and applying the same summation rule.

The interaction fingerprints are defined by flattening the matrices $\mathbf{M}_{\text{index}}$ and $\mathbf{M}_{\text{AA}}$ into vectors. These vectors are then binarized, resulting in index-based or residue type-based interaction fingerprints, respectively. Detailed information on the construction of these fingerprints is provided in the *Supporting Information.*

### Calculation of similarity matrices based on index-based fingerprints

We defined the similarity between two interaction fingerprints, $\mathbf{fp}_i$ and $\mathbf{fp}_j$, as

$$s_{i,j} = \frac{\sum_{\text{all bits}}(\mathbf{fp}_i \wedge \mathbf{fp}_j)}{\sum_{\text{all bits}}(\mathbf{fp}_i \vee \mathbf{fp}_j)}. \tag{1}$$

The operators $\wedge$ and $\vee$ represent the bitwise AND and OR operations between binary fingerprints, respectively. The above definition is often referred as Tanimoto similarity for binary vectors. For each ligand molecule in a simulation system, we compute a similarity matrix in which each element represents the similarity between condensate-ligand conformations at two distinct simulation snapshots.

### Definition of single-chain dominance

For a particular binding pose, the single-chain dominance, $f_{\text{SCD}}$, is computed from the interaction fingerprint as

$$f_{\text{SCD}} = \frac{\max(N_1, ..., N_m)}{\sum_{i=1}^{m} N_i}, \tag{2}$$

where $m$ is the number of protein chains interacting with the small molecule, and $N_i$ is the number of interactions between the small molecule and the $i$-th chain.

### UMAP analysis on residue type-based fingerprints

We employed UMAP for dimensionality reduction of interaction fingerprints. Specifically, dimensionality reduction was performed on amino acid type-based interaction fingerprints using Python library `umap-learn`.[63] The Jaccard distance, calculated as 1 minus the Tanimoto similarity, was used to quantify the distance between pairs of interaction fingerprints. Default values were maintained for the remaining UMAP hyperparameters.

### Identification of small molecule interaction fragments

For a frame collected from each simulation, the location on a small molecule drug and the amino acid residues it interacted with in that frame was binned and counted. The sites contacted by protein molecules on a small molecule were summed and histograms were computed to identify locations with the highest probability of interacting with MED1, HP1$\alpha$, or NPM1 proteins in each simulation yielding atoms central to each contact. These atoms could then be used to a chemical environment using the `Chem.FindAtomEnvironmentOfRadiusN` function implemented in RDKit[79] a radius of 2 around each atom site, to construct the fragments shown in Figure 5A. These contact sites could then be used to perform a substructure search using the `HasSubstructMatch` as implemented in RDKit in order to identify chemical probes present in Kilgore et al.[28], that either had (+) or did not have (-) the indicated chemical moiety.

### Acknowledgement

This work was supported by the National Institutes of Health (Grant R35GM133580). H.R.K acknowledges support from the Damon Runyon Cancer Research Foundation Fellowship.

**Competing interests:** Authors declare that they have no competing interests.

**Data and materials availability:** Data presented in this study is available upon reasonable request to the corresponding author.

## References

(1) Banani, S. F.; Lee, H. O.; Hyman, A. A.; Rosen, M. K. Biomolecular condensates: organizers of cellular biochemistry. *Nature reviews Molecular cell biology* **2017**, *18*, 285–298.

(2) Latham, A. P.; Zhang, B. Molecular determinants for the layering and coarsening of biological condensates: Special Issue: Emerging Investigators. *Aggregate* **2022**, *3*, e306.

(3) Sabari, B. R.; Dall'Agnese, A.; Young, R. A. Biomolecular condensates in the nucleus. *Trends in biochemical sciences* **2020**, *45*, 961–977.

(4) Uversky, V. N. Intrinsically disordered proteins in overcrowded milieu: Membrane-less organelles, phase separation, and intrinsic disorder. *Current opinion in structural biology* **2017**, *44*, 18–30.

(5) Banani, S. F.; Rice, A. M.; Peeples, W. B.; Lin, Y.; Jain, S.; Parker, R.; Rosen, M. K. Compositional control of phase-separated cellular bodies. *Cell* **2016**, *166*, 651–663.

(6) Ranganathan, S.; Shakhnovich, E. I. Dynamic metastable long-living droplets formed by sticker-spacer proteins. *eLife* **2020**, *9*, e56159.

(7) Harmon, T. S.; Holehouse, A. S.; Rosen, M. K.; Pappu, R. V. Intrinsically disordered linkers determine the interplay between phase separation and gelation in multivalent proteins. *eLife* **2017**, *6*, e30294.

(8) Ries, R. J.; Zaccara, S.; Klein, P.; Olarerin-George, A.; Namkoong, S.; Pickering, B. F.; Patil, D. P.; Kwak, H.; Lee, J. H.; Jaffrey, S. R. m6A enhances the phase separation potential of mRNA. *Nature* **2019**, *571*, 424–428.

(9) Langdon, E. M.; Qiu, Y.; Ghanbari Niaki, A.; McLaughlin, G. A.; Weidmann, C. A.; Gerbich, T. M.; Smith, J. A.; Crutchley, J. M.; Termini, C. M.; Weeks, K. M., et al.

mRNA structure determines specificity of a polyQ-driven phase separation. *Science* **2018**, *360*, 922–927.

(10) Wang, J.; Choi, J. M.; Holehouse, A. S.; Lee, H. O.; Zhang, X.; Jahnel, M.; Maharana, S.; Lemaitre, R.; Pozniakovsky, A.; Drechsel, D.; Poser, I.; Pappu, R. V.; Alberti, S.; Hyman, A. A. A Molecular Grammar Governing the Driving Forces for Phase Separation of Prion-like RNA Binding Proteins. *Cell* **2018**, *174*, 688–699.e16.

(11) Mittag, T.; Pappu, R. V. A conceptual framework for understanding phase separation and addressing open questions and challenges. *Molecular cell* **2022**, *82*, 2201–2214.

(12) Riback, J. A.; Zhu, L.; Ferrolino, M. C.; Tolbert, M.; Mitrea, D. M.; Sanders, D. W.; Wei, M.-T.; Kriwacki, R. W.; Brangwynne, C. P. Composition-dependent thermodynamics of intracellular phase separation. *Nature* **2020**, *581*, 209–214.

(13) Hnisz, D.; Shrinivas, K.; Young, R. A.; Chakraborty, A. K.; Sharp, P. A. A phase separation model for transcriptional control. *Cell* **2017**, *169*, 13–23.

(14) Klein, I. A.; Boija, A.; Afeyan, L. K.; Hawken, S. W.; Fan, M.; Dall'Agnese, A.; Oksuz, O.; Henninger, J. E.; Shrinivas, K.; Sabari, B. R., et al. Partitioning of cancer therapeutics in nuclear condensates. *Science* **2020**, *368*, 1386–1392.

(15) Boeynaems, S.; Alberti, S.; Fawzi, N. L.; Mittag, T.; Polymenidou, M.; Rousseau, F.; Schymkowitz, J.; Shorter, J.; Wolozin, B.; Van Den Bosch, L., et al. Protein phase separation: a new phase in cell biology. *Trends in cell biology* **2018**, *28*, 420–435.

(16) Shin, Y.; Brangwynne, C. P. Liquid phase condensation in cell physiology and disease. *Science* **2017**, *357*, eaaf4382.

(17) Boija, A.; Klein, I. A.; Young, R. A. Biomolecular condensates and cancer. *Cancer cell* **2021**, *39*, 174–192.

(18) Alberti, S.; Hyman, A. A. Biomolecular condensates at the nexus of cellular stress, protein aggregation disease and ageing. *Nature reviews Molecular cell biology* **2021**, *22*, 196–213.

(19) Kilgore, H. R.; Young, R. A. Learning the chemical grammar of biomolecular condensates. *Nature chemical biology* **2022**, *18*, 1298–1306.

(20) Wang, W.; Chen, Y.; Xu, A.; Cai, M.; Cao, J.; Zhu, H.; Yang, B.; Shao, X.; Ying, M.; He, Q. Protein phase separation: A novel therapy for cancer? *British Journal of Pharmacology* **2020**, *177*, 5008–5030.

(21) Zbinden, A.; Pérez-Berlanga, M.; De Rossi, P.; Polymenidou, M. Phase separation and neurodegenerative diseases: a disturbance in the force. *Developmental cell* **2020**, *55*, 45–68.

(22) Alberti, S.; Dormann, D. Liquid–liquid phase separation in disease. *Annual review of genetics* **2019**, *53*, 171–194.

(23) Mensah, M. A. et al. Aberrant Phase Separation and Nucleolar Dysfunction in Rare Genetic Diseases. *Nature* **2023**,

(24) Banani, S. F.; Afeyan, L. K.; Hawken, S. W.; Henninger, J. E.; Dall'Agnese, A.; Clark, V. E.; Platt, J. M.; Oksuz, O.; Hannett, N. M.; Sagi, I.; Lee, T. I.; Young, R. A. Genetic Variation Associated with Condensate Dysregulation in Disease. *Developmental Cell* **2022**, *57*, 1776–1788.e8.

(25) Tsang, B.; Pritišanac, I.; Scherer, S. W.; Moses, A. M.; Forman-Kay, J. D. Phase Separation as a Missing Mechanism for Interpretation of Disease Mutations. *Cell* **2020**, *183*, 1742–1756.

(26) Mitrea, D. M.; Mittasch, M.; Gomes, B. F.; Klein, I. A.; Murcko, M. A. Modulating biomolecular condensates: a novel approach to drug discovery. *Nature Reviews Drug Discovery* **2022**, *21*, 841–862.

(27) Li, S.; Wang, Y.; Lai, L. Small molecules in regulating protein phase separation: Regulating protein phase separation by small molecules. *Acta Biochimica et Biophysica Sinica* **2023**, *55*, 1075.

(28) Kilgore, H. R.; Mikhael, P. G.; Overholt, K. J.; Boija, A.; Hannett, N. M.; Van Dongen, C.; Lee, T. I.; Chang, Y.-T.; Barzilay, R.; Young, R. A. Distinct chemical environments in biomolecular condensates. *Nature Chemical Biology* **2024**, *20*, 291–301.

(29) Thody, S. A.; Clements, H. D.; Baniasadi, H.; Lyon, A. S.; Sigman, M. S.; Rosen, M. K. Small molecule properties define partitioning into biomolecular condensates. *BioRxiv* **2022**, 2022–12.

(30) Dumelie, J. G.; Chen, Q.; Miller, D.; Attarwala, N.; Gross, S. S.; Jaffrey, S. R. Biomolecular Condensates Create Phospholipid-Enriched Microenvironments. *Nature Chemical Biology*

(31) Du, X.; Li, Y.; Xia, Y.-L.; Ai, S.-M.; Liang, J.; Sang, P.; Ji, X.-L.; Liu, S.-Q. Insights into protein–ligand interactions: mechanisms, models, and methods. *International journal of molecular sciences* **2016**, *17*, 144.

(32) Sousa, S. F.; Fernandes, P. A.; Ramos, M. J. Protein–ligand docking: current status and future challenges. *Proteins: structure, function, and bioinformatics* **2006**, *65*, 15–26.

(33) Gilson, M. K.; Zhou, H.-X. Calculation of protein-ligand binding affinities. *Annu. Rev. Biophys. Biomol. Struct.* **2007**, *36*, 21–42.

(34) Wang, R.; Lai, L.; Wang, S. Further development and validation of empirical scoring functions for structure-based binding affinity prediction. *Journal of computer-aided molecular design* **2002**, *16*, 11–26.

(35) Borcherds, W.; Bremer, A.; Borgia, M. B.; Mittag, T. How do intrinsically disordered protein regions encode a driving force for liquid–liquid phase separation? *Current opinion in structural biology* **2021**, *67*, 41–50.

(36) Hyman, A. A.; Weber, C. A.; Jülicher, F. Liquid-liquid phase separation in biology. *Annual review of cell and developmental biology* **2014**, *30*, 39–58.

(37) Åqvist, J.; Luzhkov, V. B.; Brandsdal, B. O. Ligand binding affinities from MD simulations. *Accounts of chemical research* **2002**, *35*, 358–365.

(38) Guterres, H.; Im, W. Improving protein-ligand docking results with high-throughput molecular dynamics simulations. *Journal of Chemical Information and Modeling* **2020**, *60*, 2189–2198.

(39) Ye, S.; Latham, A. P.; Tang, Y.; Hsiung, C.-H.; Chen, J.; Luo, F.; Liu, Y.; Zhang, B.; Zhang, X. Micropolarity governs the structural organization of biomolecular condensates. *Nature chemical biology* **2024**, *20*, 443–451.

(40) Latham, A. P.; Zhang, B. Consistent force field captures homologue-resolved hp1 phase separation. *Journal of chemical theory and computation* **2021**, *17*, 3134–3144.

(41) Kaur, T.; Raju, M.; Alshareedah, I.; Davis, R. B.; Potoyan, D. A.; Banerjee, P. R. Sequence-encoded and composition-dependent protein-RNA interactions control multiphasic condensate morphologies. *Nature communications* **2021**, *12*, 872.

(42) Boeynaems, S.; Holehouse, A. S.; Weinhardt, V.; Kovacs, D.; Van Lindt, J.; Larabell, C.; Van Den Bosch, L.; Das, R.; Tompa, P. S.; Pappu, R. V., et al. Spontaneous driving forces give rise to protein- RNA condensates with coexisting phases and complex material properties. *Proceedings of the National Academy of Sciences* **2019**, *116*, 7889–7898.

(43) Zheng, W.; Dignon, G. L.; Jovic, N.; Xu, X.; Regy, R. M.; Fawzi, N. L.; Kim, Y. C.; Best, R. B.; Mittal, J. Molecular details of protein condensates probed by microsecond long atomistic simulations. *The Journal of Physical Chemistry B* **2020**, *124*, 11671–11679.

(44) Benayad, Z.; von Bülow, S.; Stelzl, L. S.; Hummer, G. Simulation of FUS protein condensates with an adapted coarse-grained model. *Journal of chemical theory and computation* **2020**, *17*, 525–537.

(45) Espinosa, J. R.; Joseph, J. A.; Sanchez-Burgos, I.; Garaizar, A.; Frenkel, D.; Collepardo-Guevara, R. Liquid Network Connectivity Regulates the Stability and Composition of Biomolecular Condensates with Many Components. *Proceedings of the National Academy of Sciences of the United States of America 117*, 13238–13247.

(46) Das, S.; Amin, A. N.; Lin, Y. H.; Chan, H. S. Coarse-Grained Residue-Based Models of Disordered Protein Condensates: Utility and Limitations of Simple Charge Pattern Parameters. *Physical Chemistry Chemical Physics 20*, 28558–28574.

(47) Sarthak, K.; Winogradoff, D.; Ge, Y.; Myong, S.; Aksimentiev, A. Benchmarking Molecular Dynamics Force Fields for All-Atom Simulations of Biological Condensates. *Journal of Chemical Theory and Computation 19*, 3721–3740.

(48) Saar, K. L.; Qian, D.; Good, L. L.; Morgunov, A. S.; Collepardo-Guevara, R.; Best, R. B.; Knowles, T. P. J. Theoretical and Data-Driven Approaches for Biomolecular Condensates. *Chemical Reviews 123*, 8988–9009.

(49) Zhang, Y.; Xu, B.; Weiner, B. G.; Meir, Y.; Wingreen, N. S. Decoding the Physical Principles of Two-Component Biomolecular Phase Separation. *eLife 10*, 1–31.

(50) Statt, A.; Casademunt, H.; Brangwynne, C. P.; Panagiotopoulos, A. Z. Model for Disordered Proteins with Strongly Sequence-Dependent Liquid Phase Behavior. *The Journal of Chemical Physics 152*, 075101.

(51) Latham, A. P.; Zhang, B. On the Stability and Layered Organization of Protein-DNA Condensates. *Biophysical Journal 121*, 1727–1737.

(52) Liu, S.; Wang, C.; Latham, A. P.; Ding, X.; Zhang, B. OpenABC enables flexible, simplified, and efficient GPU accelerated simulations of biomolecular condensates. *PLoS Computational Biology* **2023**, *19*, e1011442.

(53) Latham, A. P.; Zhu, L.; Sharon, D. A.; Ye, S.; Willard, A. P.; Zhang, X.; Zhang, B. Microphase Separation Produces Interfacial Environment within Diblock Biomolecular Condensates. *eLife* **2024**, *12*.

(54) Ingólfsson, H. I.; Rizuan, A.; Liu, X.; Mohanty, P.; Souza, P. C.; Marrink, S. J.; Bowers, M. T.; Mittal, J.; Berry, J. Multiscale simulations reveal TDP-43 molecular-level interactions driving condensation. *Biophysical Journal* **2023**, *122*, 4370–4381.

(55) Latham, A. P.; Zhang, B. Maximum entropy optimized force field for intrinsically disordered proteins. *Journal of chemical theory and computation* **2019**, *16*, 773–781.

(56) Souza, P. C.; Alessandri, R.; Barnoud, J.; Thallmair, S.; Faustino, I.; Grünewald, F.; Patmanidis, I.; Abdizadeh, H.; Bruininks, B. M.; Wassenaar, T. A., et al. Martini 3: a general purpose force field for coarse-grained molecular dynamics. *Nature methods* **2021**, *18*, 382–388.

(57) Huang, J.; Rauscher, S.; Nawrocki, G.; Ran, T.; Feig, M.; De Groot, B. L.; Grubmüller, H.; MacKerell Jr, A. D. CHARMM36m: an improved force field for folded and intrinsically disordered proteins. *Nature methods* **2017**, *14*, 71–73.

(58) Deng, Z.; Chuaqui, C.; Singh, J. Structural interaction fingerprint (SIFt): a novel method for analyzing three-dimensional protein- ligand binding interactions. *Journal of medicinal chemistry* **2004**, *47*, 337–344.

(59) Pérez-Nueno, V. I.; Rabal, O.; Borrell, J. I.; Teixidó, J. APIF: a new interaction fingerprint based on atom pairs and its application to virtual screening. *Journal of chemical information and modeling* **2009**, *49*, 1245–1260.

(60) Gainza, P.; Sverrisson, F.; Monti, F.; Rodola, E.; Boscaini, D.; Bronstein, M. M.; Correia, B. E. Deciphering interaction fingerprints from protein molecular surfaces using geometric deep learning. *Nature Methods* **2020**, *17*, 184–192.

(61) Marcou, G.; Rognan, D. Optimizing fragment and scaffold docking by use of molecular interaction fingerprints. *Journal of chemical information and modeling* **2007**, *47*, 195–207.

(62) Bouysset, C.; Fiorucci, S. ProLIF: a library to encode molecular interactions as fingerprints. *Journal of Cheminformatics* **2021**, *13*, 72.

(63) McInnes, L.; Healy, J.; Melville, J. Umap: Uniform manifold approximation and projection for dimension reduction. *arXiv preprint arXiv:1802.03426* **2018**,

(64) Dumelie, J. G.; Chen, Q.; Miller, D.; Attarwala, N.; Gross, S. S.; Jaffrey, S. R. Biomolecular condensates create phospholipid-enriched microenvironments. *Nature Chemical Biology* **2024**, *20*, 302–313.

(65) Dignon, G. L.; Best, R. B.; Mittal, J. Biomolecular phase separation: from molecular driving forces to macroscopic properties. *Annual review of physical chemistry* **2020**, *71*, 53–75.

(66) Burke, K. A.; Janke, A. M.; Rhine, C. L.; Fawzi, N. L. Residue-by-residue view of in vitro FUS granules that bind the C-terminal domain of RNA polymerase II. *Molecular cell* **2015**, *60*, 231–241.

(67) Abraham, M. J.; Murtola, T.; Schulz, R.; Páll, S.; Smith, J. C.; Hess, B.; Lindahl, E.

GROMACS: High performance molecular simulations through multi-level parallelism from laptops to supercomputers. *SoftwareX* **2015**, *1*, 19–25.

(68) Souza, P. C. et al. Martini 3: A General Purpose Force Field for Coarse-Grained Molecular Dynamics. *Nature Methods 18*, 382–388.

(69) Li, Y.; Zhang, Y. REMO: A new protocol to refine full atomic protein models from C-alpha traces by optimizing hydrogen-bonding networks. *Proteins: Structure, Function, and Bioinformatics* **2009**, *76*, 665–676.

(70) Kroon, P. C.; Grünewald, F.; Barnoud, J.; van Tilburg, M.; Souza, P. C.; Wassenaar, T. A.; Marrink, S.-J. Martinize2 and vermouth: Unified framework for topology generation. *arXiv preprint arXiv:2212.01191* **2022**,

(71) Wassenaar, T. A.; Pluhackova, K.; Bockmann, R. A.; Marrink, S. J.; Tieleman, D. P. Going backward: a flexible geometric approach to reverse transformation from coarse grained to atomistic models. *Journal of chemical theory and computation* **2014**, *10*, 676–690.

(72) Vanommeslaeghe, K.; MacKerell Jr, A. D. Automation of the CHARMM General Force Field (CGenFF) I: bond perception and atom typing. *Journal of chemical information and modeling* **2012**, *52*, 3144–3154.

(73) Vanommeslaeghe, K.; Raman, E. P.; MacKerell Jr, A. D. Automation of the CHARMM General Force Field (CGenFF) II: assignment of bonded parameters and partial atomic charges. *Journal of chemical information and modeling* **2012**, *52*, 3155–3168.

(74) Hess, B.; Bekker, H.; Berendsen, H. J.; Fraaije, J. G. LINCS: A linear constraint solver for molecular simulations. *Journal of computational chemistry* **1997**, *18*, 1463–1472.

(75) Hopkins, C. W.; Le Grand, S.; Walker, R. C.; Roitberg, A. E. Long-time-step molecular dynamics through hydrogen mass repartitioning. *Journal of chemical theory and computation* **2015**, *11*, 1864–1874.

(76) Sehnal, D.; Bittrich, S.; Deshpande, M.; Svobodová, R.; Berka, K.; Bazgier, V.; Velankar, S.; Burley, S. K.; Koča, J.; Rose, A. S. Mol* Viewer: modern web app for 3D visualization and analysis of large biomolecular structures. *Nucleic acids research* **2021**, *49*, W431–W437.

(77) Michaud-Agrawal, N.; Denning, E. J.; Woolf, T. B.; Beckstein, O. MDAnalysis: a toolkit for the analysis of molecular dynamics simulations. *Journal of computational chemistry* **2011**, *32*, 2319–2327.

(78) McGibbon, R. T.; Beauchamp, K. A.; Harrigan, M. P.; Klein, C.; Swails, J. M.; Hernández, C. X.; Schwantes, C. R.; Wang, L.-P.; Lane, T. J.; Pande, V. S. MDTraj: a modern open library for the analysis of molecular dynamics trajectories. *Biophysical journal* **2015**, *109*, 1528–1532.

(79) Landrum, G., et al. RDKit: A software suite for cheminformatics, computational chemistry, and predictive modeling. **2013**, `https://www.rdkit.org/RDKit_Overview.pdf`.

## Supplementary Files

This is a list of supplementary files associated with this preprint. Click to download.

- suppinforCWBZ.pdf
